# Predictive value of Albumin-Bilirubin grade for intravenous immunoglobulin resistance in a large cohort of patients with Kawasaki disease: a prospective study

**DOI:** 10.1186/s12969-021-00638-7

**Published:** 2021-09-25

**Authors:** Yu Yan, Lina Qiao, Yimin Hua, Shuran Shao, Nanjun Zhang, Mei Wu, Lei Liu, Kaiyu Zhou, Xiaoliang Liu, Chuan Wang

**Affiliations:** 1 Department of Pediatric Cardiology, West China Second University Hospital, Sichuan University, 610041 Chengdu, Sichuan China; 2 West China Medical School of Sichuan University, 610041 Chengdu, Sichuan China; 3 Key Laboratory of Birth Defects and Related Diseases of Women and Children (Sichuan University), Ministry of Education, Sichuan 610041 Chengdu, China; 4 Key Laboratory of Development and Diseases of Women and Children of Sichuan Province, West China Second University Hospital, Sichuan University, 610041 Chengdu, Sichuan China; 5 The Cardiac development and early intervention unit, West China Second University Hospital, West China Institute of Women and Children’s Health, Sichuan University, 610041 Chengdu, Sichuan China; 6 Department of Pediatrics, West China Second University Hospital, Sichuan University, 610041 Chengdu, Sichuan China; 7 Dept. of Pediatrics, West China Second University Hospital, Sichuan University, No. 20, 3rd section, South Renmin Road, 610041 Chengdu, China

**Keywords:** Kawasaki disease, albumin-bilirubin grade, intravenous immunoglobulin resistance

## Abstract

**Background:**

Intravenous immunoglobulin (IVIG) resistance prediction is one of the primary clinical issues and study hotspots in KD. This study aimed to prospectively investigate the value of albumin-bilirubin grade (ALBI) in predicting IVIG resistance in KD and to assess whether ALBI has more predictive value or accuracy than either ALB or TBil alone in predicting IVIG resistance.

**Methods:**

A total of 823 patients with KD were prospectively enrolled. The clinical and laboratory data were compared between the IVIG-response group (*n* = 708) and the IVIG-resistance group (*n* = 115). Multivariate logistic regression analysis was performed to identify the independent risk factors for IVIG resistance. Receiver operating characteristic (ROC) curves analysis was applied to assess the validity of ALBI, ALB, and TBil in predicting IVIG resistance.

**Results:**

ALBI was significantly higher in patients with IVIG resistance and was identified as an independent risk factor for IVIG resistance in KD. The parameter of ALBI ≥ − 2.57 (AUC: 0.705, 95 %CI: 0.672–0.736), ALB ≤ 33.0 g/L (AUC: 0.659, 95 %CI: 0.626–0.692), and TBil ≥ 16.0µmol/L (AUC: 0.626, 95 %CI: 0.592–0.659), produced a sensitivity, specificity, PPV, and NPV of 0.617, 0.657, 0.226 and 0.914; 0.374, 0.850, 0.289 and 0.893; 0.269, 0.941, 0.425 and 0.888, respectively.

**Conclusions:**

A higher ALBI was an independent risk factor for IVIG resistance in KD. It yielded better predictive ability than ALB and TBil alone for initial IVIG resistance.

**Supplementary Information:**

The online version contains supplementary material available at 10.1186/s12969-021-00638-7.

## Introduction

Kawasaki disease (KD) is an acute systemic vasculitis predominantly affecting children, with coronary artery lesions (CALs) as the most severe sequela [1]. However, approximately 10–20 % of patients with KD are resistant to intravenous immunoglobulin(IVIG) treatment and develop a risk of CALs [2] despite timely IVIG treatment is substantially effective. It has been proven that IVIG-resistant patients may benefit from adjunctive therapies for primary treatment, including corticosteroids [3, 4], infliximab [5, 6], plasma exchange [7, 8], and cytotoxic agents [9, 10]. Thus, early prediction of IVIG resistance is of paramount importance since they may benefit from early-intensified therapy. Although several risk-scoring systems have been developed by Kobayashi, Egami, and Sano in Japan [11–13], these systems are considered to be complicated and unpractical as multiple points need to be summed showing variable prediction effectiveness for IVIG resistance in different populations [14–22].

Despite the definite cause of KD is currently unknown, it is generally accepted that systematic inflammatory response plays a crucial role in the pathogenesis of the onset and progression of KD [23]. The innate immune system is activated as an early event of KD onset, characterized by increasing concentration of various cytokines in circulating blood such as interleukin-1(IL-1), IL-6, IL-8, and tumor necrosis factor-α (TNF-α) [23, 24]. The serum albumin (ALB), traditionally regarded as a maker of nutritional status, is also increasingly considered as the most important negative acute-phase protein [25]. Catabolism of ALB is directly correlated with the severity of the acute inflammation. Hypoalbuminemia is commonly observed in patients with KD, primarily resulting from the increased permeability and leakage of serum ALB during the acute phase [26, 27]. Meanwhile, serum total bilirubin is constantly elevated and could reflect the degree of liver damage in KD. Substantial evidence proved that both hypoalbuminemia and hyperbilirubinemia were associated with IVIG resistance [11–13, 28, 29]. However, neither was suitable alone to be a valuable predictor without high sensitivities or specificities. Even though both indicators were included in several risk-scoring systems for IVIG resistance prediction in KD [13, 28–32], these risk-scoring systems are based on individual parameters that are scored based on arbitrarily defined predetermined cut-off values, producing variable predictive ability for IVIG resistance [14–16]. Inevitably, these risk-scoring systems or single indicators stratified patients into distinct groups, resulting in information loss. Notably, patients who fall around the cut-off point (i.e., just below or above the discriminating value) may be classified as having different risk levels. Therefore, the predictive ability of ALB, TBil, or available risk-scoring systems comprising ALB and/or TBil, seems to be limited and unsatisfactory for IVIG resistance. It indicated the necessity and importance of developing a useful and stable predictor for initial IVIG resistance.

Recently, a parameter known as the ALBI grade [0.66×log_10_^TBil^-0.085×ALB] provides information on ALB and TBil, reflecting systemic inflammatory response and/or the degree of liver damage [33]. ALBI has been proposed as a powerful prognostic indicator of poor outcomes in patients with hepatocellular carcinoma (HCC) [33–37], hepatitis virus infection, and acute pancreatitis [38–40]. Additionally, this parameter positively correlates with inflammatory markers [39]. However, the usefulness of ALBI as a predictor of prognosis has not been evaluated in patients with KD. Thus, in this cohort, we prospectively assessed the predictive ability of ALBI for IVIG resistance in patients with KD and whether ALBI has more predictive value or accuracy than either ALB or TBil alone in predicting IVIG resistance.

## Patients and methods

Patients with KD were prospectively recruited between March 2015 and September 2020 at our hospital. KD was diagnosed based on the AHA scientific statement’s standards for diagnosis, treatment, and long-term management of KD [1] and confirmed by two experienced pediatricians (including ≥ 1 KD specialist). The classic diagnosis of KD has been based on the presence of ≥ 5 days of fever and ≥ 4 of the five principal clinical features (Rash, Extremity changes, Conjunctivitis, Oral changes, Cervical lymphadenopathy) while the diagnosis of incomplete KD included prolonged unexplained fever, fewer than 4 of the principal clinical findings, and compatible laboratory or echocardiographic findings [1]. Structured questionnaires with pre-coded questions, including basic demographic information, clinical manifestations, hematological examination results, treatment, and follow-up outcomes, were used for data collection. All questionnaires were pretested and revised accordingly. Two well-trained doctors conducted data collection and checked the questionnaires to ensure completeness. Informed written consent was obtained from the parents after this study’s nature was fully explained to them. The University Ethics Committee approved the study on Human Subjects at Sichuan University. All research was performed following the relevant guidelines and regulations.

**Fig. 1:**
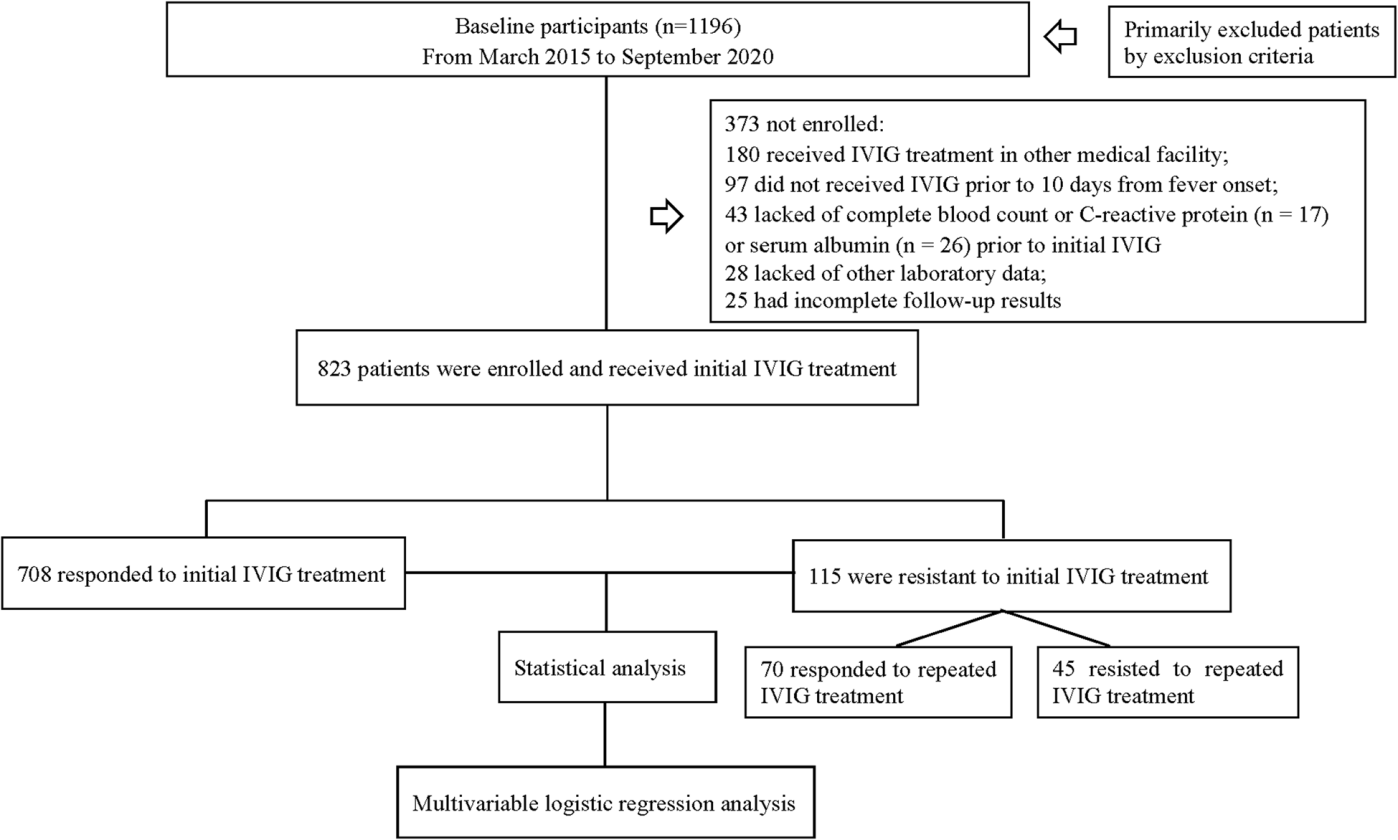
The flowchart of our prospective cohort study

Exclusion criteria included known patients with congenital or chronic liver disease affecting ALB and/or TBil levels. In total, 1196 patients were diagnosed with KD upon admission. Those who had received IVIG treatment in other medical facilities (*n* = 180) or did not receive IVIG treatment before 10 days from fever onset (*n* = 97) were excluded. Additionally, 43 patients were excluded due to lack of data regarding complete blood count (CBC) or C-reactive protein(CRP) (*n* = 17) or serum ALB (*n* = 26) levels prior to initial IVIG treatment. We also excluded 53 patients because other laboratory data (*n* = 28) or follow-up results (*n* = 25) were incomplete. Finally, 823 patients were enrolled for analysis, including 708 initial IVIG responders and 115 initial IVIG non-responders. Of the 115 patients with initial IVIG resistance, 45 did not respond to repeated IVIG treatment and received pulse intravenous methylprednisolone infusion (Fig. 1). No patients received additional treatment such as infliximab, plasma exchange, or cytotoxic agents. The ALBI grade was defined as 0.66×log_10_^TBil^-0.085×ALB before the initial IVIG infusion. In case of more than one TBil or ALB determination before the initial IVIG infusion, the highest value of TBil and the lowest value of ALB were chosen for the analysis.

All patients received the same standard treatment regimen for KD. Aspirin (30–50 mg/kg/day) and IVIG (2 g/kg given as a single intravenous infusion) were administered within the first ten days of illness from fever onset. After patients were treated for 48–72 h, a tapered dose of aspirin (3–5 mg/kg/day) was administered for 6–8 weeks. If patients had CALs, aspirin was continued until there was no evidence of CALs. The initial IVIG resistance was defined as recurrent or persistent fever (≥ 38.0 °C) or other clinical signs of KD for at least 36 h but not longer than seven days after initial IVIG [41]. A second IVIG (2 g/kg given as a single intravenous infusion) was administered if the patient had initial IVIG resistance. Furthermore, if the patient had repeated IVIG resistance defined as having recurrent or persistent fever after the second IVIG infusion, tapered administration of pulse intravenous methylprednisolone (20–30 mg/kg/day for three consecutive days) followed by oral prednisone (2 mg/kg/day) for seven days was given as adjunctive therapy.

During the acute and sub-acute phase of KD [42, 43], CALs were based on the normalization of dimensions for body surface area (BSA) as Z scores (standard deviation units from the mean, normalized for BSA) by the AHA scientific statement’s standards for diagnosis, treatment, and long-term management of KD [44]. According to the institutional protocol, patients underwent standardized echocardiograms by two pediatric ultrasonologists during the acute phase and 6–8 weeks during the cardiology clinic follow-up evaluations until the resolution of CALs. BSA and Z scores were calculated using the Haycock [45] and the Kobayashi equations [46], respectively.

### Statistical analyses

Categorical variables are described as frequencies and percentages (n/%) where appropriate, and quantitative data are presented as the median with the 25th and 75th percentiles (interquartile range (IQR)) in square brackets. The Shapiro-Wilk test and homogeneity test of variance were used to confirm that quantitative data from different groups were normally distributed and met the homogeneity of variance. The chi-squared test, unpaired Student’s t-test, and Mann-Whitney U test were applied to compare demographic characteristics, clinical manifestations, and laboratory data between groups. Numerical variables that showed statistical significance in the univariate analysis were transformed into dichotomous variables. Cut-off values corresponding to the maximum Youden’s index for sensitivity and specificity were selected based on the receiver operating characteristic (ROC) curve. These crucial indicators from univariate analysis were then subjected to multivariate logistic regression analysis to identify independent predictors of IVIG resistance. The best cut-off values of the multivariable model for IVIG resistance prediction and its corresponding predictive power were further assessed using the ROC curve. The parameters of TBil and ALB were not included in the multivariate logistic regression analysis because of their strong correlations with ALBI. To compare the predictive value of ALBI and ALB or TBil for IVIG resistance, ROC analysis was conducted to determine the best cut-off values and their corresponding predictive validities. Sensitivity, specificity, positive predictive value (PPV), negative predictive value (NPV), and diagnostic accuracy were assessed. A De Long test was used to compare the ROC curves. P values < 0.05 were considered statistically significant. All data analyses were performed using SPSS 21.0 (IBM SPSS Statistics version 21.0, Armonk, NY, IBM Corp.).

## Results

### Comparison of clinical data between initial IVIG-response group and IVIG-resistance group

As shown in Table 1, no significant differences were observed between the two groups regarding sex, fever duration before IVIG treatment, the occurrence of incomplete KD, and typical clinical manifestations of KD (*p* > 0.05). The incidence of CALs was relatively higher in the IVIG-resistance group, but the difference was not statistically significant (13.0 % vs. 10.0 %, *p* = 0.325). Compared with patients from the IVIG-response group, patients from the IVIG-resistant group were older, presenting substantially higher levels of neutrophil percentage (N%), alanine aminotransferase (ALT), TBil, and ALBI, but lower levels of hemoglobin, platelet (PLT), ALB and sodium (Na^+^) (*p* < 0.005).

**Table 1:** Comparison of clinical data between the groups of initial IVIG-response and IVIG-resistance in KD

	IVIG-response (*n* = 708)	IVIG-resistance (*n* = 115)	*p* value
Male	403(56.9)	59(51.3)	0.267
Age, years	2.1(1.2–3.6)	2.3(1.3–4.5)	0.026
**Clinical manifestations**			
Rash	549(77.5)	96(83.5)	0.190
Extremity changes	393(55.5)	65(57.4)	0.762
Conjunctivitis	651(91.9)	103(89.6)	0.368
Oral changes	639(90.3)	108(93.9)	0.296
Cervical lymphadenopathy	310(43.8)	62(53.9)	0.055
Fever duration before initial IVIG, days	5.0(5.0–6.0)	5.0(4.0–6.0)	0.212
Incomplete KD	275(38.8)	36(31.3)	0.146
CALs in the acute phase of KD	71(10.0)	15(13.0)	0.325
**Before initial IVIG**			
Time of blood test from fever onset, days	5.0(4.0–6.0)	4.0(3.0–5.0)	0.558
WBC, ×10^9^/L	13.2(10.7–16.6)	13.6(10.0-16.9)	0.515
Neutrophil, %	66.2(77.0–56.0)	77.5(66.2–84.0)	<0.001
Lymphocyte, %	24.6(16.0–33.0)	15.0(9.6–24.0)	<0.001
Hemoglobin, g/L	109(102–117)	107(101–114)	0.036
PLT, ×10^9^/L	316(259–387)	290(196–348)	<0.001
CRP, mg/L	71.0(43.0-107.0)	92.1(62.0-144.0)	<0.001
ESR, mm/h	64.0(46.0–81.0)	67.0(48.0–87.0)	0.152
AST, U/L	33.0(25.0–49.0)	37.0(25.0–73.0)	0.161
ALT, U/L	36.0(20.0-77.7)	54.0(27.0-125.0)	0.006
ALB, g/L	38.0(35.0–41.0)	36.0(30.3–39.0)	0.004
TBil, µmol/L	6.0(4.0-8.5)	7.0(5.0–19.0)	<0.001
Na^+^, mmol/L	137.0(135.0-139.0)	135.0(133.0-137.0)	<0.001
ALBI	–2.73(–3.00- (–2.46))	–2.38(–2.69- (–1.93))	<0.001

Abbreviations: *ALB* Albumin; *AST* aspartate aminotransferase; *ALT* alanine aminotransferase; *CRP* C-reactive protein; *CALs* coronary artery lesions; *ESR* erythrocyte sedimentation rate; *IVIG* intravenous immunoglobulin; *KD* Kawasaki disease; *TBil* total bilirubin; *Na*^+^, sodium; *WBC* white blood cell; *ALBI* albumin-bilirubin index;

The data are presented as the median with the 25th and 75th percentiles in square brackets for continuous variables and as the percentage for the categorical variables

### Multivariate logistic regression analysis for IVIG resistance prediction in KD

As shown in Table 2, multivariate logistic regression analysis was performed to investigate whether ALBI was an independent risk factor for IVIG resistance in KD. Statistically significant variables including age, N%, PLT, hemoglobin, CRP, ALT and Na^+^ levels were identified as confounding factors. The multivariate logistic regression model did not comprise the parameters of ALB and TBil since all of them showed strong correlations with ALBI. The results showed that ALBI ≥ − 2.57 was an independent risk factor for initial IVIG resistance (OR: 3.374, 95 %CI: 2.016–5.648, *p* < 0.001).

**Table 2:** Multivariate logistic regression analysis for predicting IVIG resistance in KD

	*β*	SE	Walds	*P*-value	OR	95 % CI
Age ≥ 4.42 years	0.265	0.286	0.855	0.355	1.303	0.744–2.284
Neutrophil ≥ 76.2 %	0.460	0.256	3.233	0.072	1.585	0.959–2.617
Platelet ≤ 312 × 10^9^/L	0.504	0.232	4.720	0.030	1.655	1.051–2.608
Hemoglobin ≤ 104 g/L	0.341	0.226	2.268	0.132	1.406	0.902–2.190
CRP ≥ 57.4 mg/L	0.268	0.278	0.934	0.334	1.308	0.759–2.253
ALT ≥ 41U/L	0.260	0.232	1.255	0.263	1.297	0.823–2.044
Na + ≤ 135.4mmol/L	0.752	0.233	10.391	0.001	2.121	1.343–3.350
ALBI≥-2.14	1.216	0.263	21.406	0.000	3.374	2.016–5.648
Interpret	-0.745	0.327	5.185	0.023	0.475	

Abbreviations: *ALBI* albumin-bilirubin index; *ALT* alanine aminotransferase; *ALB* albumin; *CRP* C-reactive protein; *IVIG* intravenous immunoglobulin; *TBil* total bilirubin; *KD* Kawasaki disease; *Na+*, serum sodium;

### Predictive ability of ALBI, ALB, and TBil in predicting initial IVIG resistance

The parameters of ALBI ≥ − 2.57, ALB ≤ 33.0 g/L, and TBil ≥ 16.0µmol/L produced a sensitivity, specificity, PPV, NPV and diagnostic accuracy of 0.617, 0.657, 0.226, 0.914, and 0.651; 0.374, 0.850, 0.289, 0.893, and 0.783; 0.269, 0.941, 0.425, 0.888, and 0.847, respectively (Table 3).

**Table 3:** Sensitivity, specificity, PPV, NPV, diagnostic accuracy of ALBI, ALB, and TBil cut-off values in initial IVIG resistance prediction

	AUC	SE	95 %CI	Sensitivity	Specificity	PPV	NPV	Diagnostic accuracy	*p* value
ALBI≥-2.57	0.705	0.0277	0.672–0.736	0.617	0.657	0.226	0.914	0.651	< 0.001
ALB ≤ 33.0 g/L*	0.659	0.0281	0.626–0.692	0.374	0.850	0.289	0.893	0.783	< 0.001
TBil ≥ 16.0µmol/L*	0.626	0.0305	0.592–0.659	0.269	0.941	0.425	0.888	0.847	< 0.001

Abbreviations: *ALB* serum albumin; *TBil*, total bilirubin; ALBI = 0.66×log_10_^TBil^-0.085×ALB; *IVIG* intravenous immunoglobulin; *NPV* negative predictive value; *PPV*, positive predictive value

Pairwise comparison of ROC curves between ALBI and ALB, TBil in predicting IVIG resistance by De Long test, *p* = 0.001 and 0.012

**Fig. 2:**
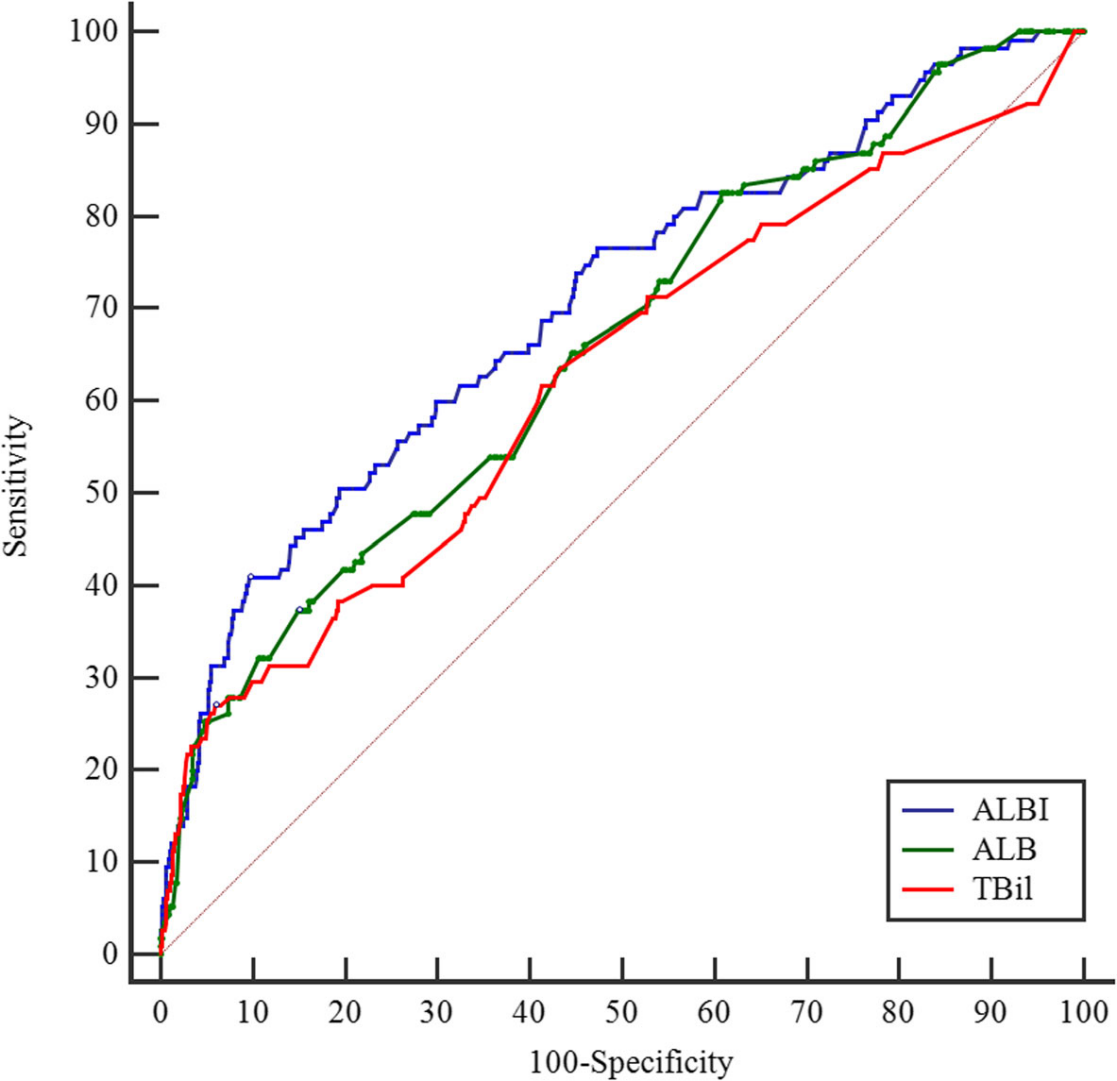
The receiver operating characteristic (ROC) curve for ALBI, ALB, and TBil in predicting initial IVIG resistance

The AUC value of ALBI (AUC: 0.705, 95 %CI: 0.672–0.736) did significantly differed from that of ALB (AUC: 0.659, 95 %CI: 0.626–0.692) and TBil (AUC: 0.626, 95 %CI: 0.592–0.659) (*p* = 0.001 and 0.012, respectively) (Fig. 2). The diagnostic sensitivity and specificity of ALBI, TBil, and ALB according to the ROC optimized decision limits in predicting initial IVIG resistance are shown in Table 4.

**Table 4:** Diagnostic specificity and sensitivity according to ROC-optimized decision limits for ALBI, ALB, and TBil in predicting initial IVIG resistance among patients with KD

	Estimated specificity at fixed sensitivity (*n* = 823)				Estimated sensitivity at fixed specificity (*n* = 823)			
ALBI	Sensitivity(%)	Specificity(%)	Cut-off point	n	Specificity(%)	Sensitivity(%)	Cut-off point	n
	99.0	7.9	-3.29	767	99.0	10.4	-1.58	20
	97.5	13.3	-3.18	727	97.5	13.9	-1.77	35
	95.0	17.2	-3.12	698	95.0	26.1	-1.97	66
	90.0	23.6	-3.02	648	90.0	40.9	-2.15	120
ALB								
	99.0	8.5	43.8	753	99.0	4.4	26.0	12
	97.5	12.6	42.9	713	97.5	15.9	28.8	35
	95.0	16.6	41.9	661	95.0	25.2	30.4	64
	90.0	20.1	41.9	661	90.0	30.7	31.8	93
TBil								
	99.0	1.5	1.91	816	99.0	6.9	47.9	16
	97.5	2.3	1.93	816	97.5	17.4	30.3	38
	95.0	3.5	1.96	816	95.0	24.2	18.6	65
	90.0	11.7	2.94	771	90.0	29.5	12.9	106

Abbreviations: *ALBI* albumin-bilirubin index; *ALB* albumin; *TBil* total bilirubin; *IVIG* intravenous immunoglobulin; *KD* Kawasaki disease

### Association of ALBI with repeated IVIG resistance and CALs in KD

The patients were divided into two subgroups according to whether patients with KD presented with repeated IVIG resistance: patients with repeated IVIG resistance (*n* = 45) and response to the second IVIG (*n* = 70). There was no significant difference in ALBI between patients with repeated IVIG resistance and IVIG response [ − 2.47(–2.8 - (–2.02)) vs. − 2.27(–2.64-(–1.9)), *p* = 0.081] (Supplementary material 1). The ALBI tended to be higher in the patients with CALs than those with non-CALs, but the difference was not statistically significant [ − 2.63(–2.92- (–2.19)) vs. − 2.70(–3.00- (–2.42)), *p* = 0.065] (Supplementary material 2).

## Discussion

The indicators of ALB and TBil were found to be associated with IVIG resistance and included in several risk-scoring systems for IVIG resistance prediction in KD [13, 19–23]; however, neither indicator was ideal. Except for representing liver function [47–50], the ALBI grade calculated using ALB and TBil could reflect systemic inflammatory response [39]. To the best of our knowledge, this cohort was the first to explore the predictive validity of ALBI for IVIG resistance prediction. The present study incorporated data on both ALB and TBil for IVIG resistance prediction in patients with KD and compared its predictive value with ALB and TBil. Furthermore, the sensitivity, specificity, PPV, and NPV of all three predictors were also assessed. ALBI was identified as an independent risk factor for initial IVIG resistance in KD. The discriminating cut-off value of ALBI for IVIG resistance prediction was − 2.57, with moderate sensitivity (0.617) and specificity (0.657). Moreover, the discriminating ability of ALBI was superior to that of ALB and TBil in initial IVIG resistance.

The ALBI grade was firstly proposed by Johnson et al., aiming to find a simple, evidence-based, objective and discriminatory method of assessing liver function in patients with hepatocellular carcinoma (HCC) [33]. In their study, a cox regression model based on albumin and log_10_ bilirubin was built and was found to be a prognostic predictor for HCC. Thereafter, various studies proved that higher ALBI grades were associated with poor prognosis, overall survival, and disease-free survival, as well as liver function and liver cancer stage in patients with HCC [47–50]. Moreover, a recent study [39] found that the ALBI grade could reflect the severity of systemic inflammation and it was positively correlated with the level of inflammatory markers. Consistent with previous studies that the ALBI grade was a powerful prognostic indicator in patients with the aforementioned disease, we found the ALBI grade was an independent risk factor for initial IVIG resistance in KD and its predictive ability was more valuable and accurate than either ALB or TBil alone in initial IVIG resistance prediction. Recently, several clinical trials from Japan documented addition of corticosteroid or ciclosporin therapy to standard-dose IVIG and aspirin in the primary therapy of KD reduced the initial non-response rate and decrease the incidence of CALs among high-risk patients for initial IVIG resistance predicted by Kobayashi, Sano, and Egami scores [11–13], suggesting high-risk KD patients for initial IVIG resistance might mostly benefit from aggressive therapy. Since aforementioned risk-scores have been proved to be less ideal and clinical relevance in non-Japanese population [17–22], the ALBI grade may attract clinician’s attention when developing the risk-adapted therapeutic strategy in other regions.

The prediction of IVIG resistance is one of the leading clinical issues and, consequently, one of the most extensively studies topics in KD. Researchers have previously made much attempts to identify criteria and markers for such resistance. Elevation of serum CRP, ESR, AST, ALT, TBil, and N% and decreased levels of hemoglobin, Na^+^, ALB, and PLT were commonly observed in patients with KD and reported to be associated with IVIG resistance [11–13, 28–30, 51, 52]. However, the predive abilities of the above parameters as a single marker were proved to be not good enough. In our study, after multivariate logistic analysis, except for ALBI, platelet ≤ 312 × 109/L and Na^+^ ≤ 135.4mmol/L were also identified as independent risk factors for IVIG resistance in KD. As shown in supplementary material 3, consistent with previous studies, the predictive values of platelet ≤ 312 × 109/L and Na^+^ ≤ 135.4mmol/L were not satisfactory with moderate sensitivities and specificities. In addition, several risk-scoring systems incorporating laboratory and clinical parameters, such as Kobayashi, Egami, and Sano systems [11–13], have previously developed and appeared to be fairly predictive for IVIG resistance in Japanese Children. However, they seemed to be less ideal and clinical relevance in the non-Japanese population in the US, Korea, Germany, Spain, and China [17–22]. Recently, several predictive models from China, including Formosa [29], Yang’s [28], Tang’s [30], and Hua’s [51], have been developed but also showed variable predictive effectiveness even in different areas of China [53, 54]. Actually, we have previously tested the predictive value of all these risk-scoring systems in our population. As shown in supplementary material 4, it was found that the Kobayashi, Egami, Sano, Tang’s, Yang’s, and Hua’s prediction models had a relatively high specificity of 0.727–0.950, but an extremely low sensitivity of 0.170–0.513. The performances of Formosa, Moon’s, and Fu’s systems were also not good enough owing to moderate sensitivity (0.617–0.670) and specificity (0.575–0.636). In the present study, the predictive ability of ALBI as a single maker for IVIG resistance seemed to be comparable or a little bit better compared to that of Formosa’s, Moon’s, and Fu’s systems in our population.

Based on these findings, it was evident that we could not identify all the IVIG non-responders using any of the above risk scores, including ALBI. However, as simple ratios calculated from ALB and TBil, which could be obtained from the routine blood test, ALBI appeared to be a cost-effective alternative that may provide additional information for IVIG resistance prediction in KD patients. Moreover, unlike the aforementioned risk-scoring systems, the predictive value of ALBI may be more consistent and stable since hypoalbuminemia and hyperbilirubinemia were commonly observed among different populations. Nevertheless, due to an unknown origin of KD and in light of the above findings, we speculated a prediction model combined with other specific indicators rather than clinical and routine laboratory variables might have a better performance.

Besides, it is crucial to predict the development of CALs since KD patients with CALs may develop thrombosis or stenotic lesions and are at risk of myocardial infarction, sudden death, and congestive heart failure in the future [55]. In the present study, IVIG resistance was not associated with the occurrence of CALs in KD, consistent with several previous studies [56, 57]. Standard IVIG therapy for all included participants in our study might explain the negative association between IVIG resistance and CALs. Additionally, the ALBI grade also did not differ between patients with KD with and without CALs [ − 2.63 (–2.92–(–2.19)) vs. − 2.69 (–3.00 –(–2.42)), *p* = 0.065]. Previous findings suggested that persistent chronic inflammation may be more likely associated with the development of CALs [57]. Compared with the baseline ALBI grade, fluctuations in ALBI may possess greater predictive power for CALs in patients with KD. Therefore, further studies that collect ALBI at different time points are necessary to classify its predictive ability and prognosis of CALs.

This study has some potential limitations. First, selective bias may occur as this study was performed in a single institution. Second, the findings may only apply to patients with KD receiving standardized IVIG treatment (2 g/Kg) < 10 days from fever onset. Despite the above limitations, this is the first prospective study to determine the predictive value of ALBI for IVIG resistance with a large sample size. ALBI was significantly higher in patients with IVIG resistance and an independent risk factor for IVIG resistance. Due to an unknown origin of KD and in light of the above findings, we speculate that a prediction model combined with other specific indicators rather than clinical and routine laboratory variables might have a better outcome.

## Conclusions

A higher ALBI was an independent risk factor for initial IVIG resistance, yielding better predictive ability than ALB and TBil alone for initial IVIG resistance. ALBI may provide some valuable references for clinical management in further studies.

## Supplementary information

**Additional file 1: Supplementary material 1.**

**Additional file 2: Supplementary material 2.**

**Additional file 3: Supplementary material 3.**

**Additional file 4: Supplementary material 4.**

## Data Availability

All data generated or analyzed during this study are included in this published article and the supplementary files.

## Abbreviations

AHA: American Heart Association
ALB: Albumin
ALBI: Albumin-bilirubin
ALT: Alanine aminotransferase
BSA: Body surface area
CALs: Coronary artery lesions
CBC: Complete blood count
CP: Child-Pugh
CRP: C-reactive protein
HCC: Hepatocellular carcinoma
N%: Neutrophil percentage
IL: Interleukin
IQR: Interquartile range
IVIG: Intravenous immunoglobulin
KD: Kawasaki disease
NPV: Negative predictive value
Na+: sodium
PLT: Platelet
PPV: Positive predictive value
ROC: Receiver operating characteristic
TBil: Total bilirubin
TNF: Tumor necrosis factor

## References

[1] Newburger JW, Takahashi M, Gerber MA (2004). Diagnosis, treatment, and long-term management of Kawasaki disease: a statement for health professionals from the Committee on Rheumatic Fever, Endocarditis, and Kawasaki Disease, Council on Cardiovascular Disease in the Young, American Heart Association. Pediatrics.

[2] Uehara R, Belay ED, Maddox RA (2008). Analysis of potential risk factors associated with nonresponse to initial intravenous immunoglobulin treatment among Kawasaki disease patients in Japan. Pediatric Infectious Disease Journal.

[3] Newburger JW, Sleeper LA, Mccrindle BW (2007). Randomized Trial of Pulsed Corticosteroid Therapy for Primary Treatment of Kawasaki Disease. The New England journal of medicine.

[4] Kijima Y, Kamiya T, Suzuki A, Hirose O, Manabe H. A Trial Procedure to Prevent Aneurysm Formation of the Coronary Arteries by Steroid Pulse Therapy in Kawasaki Disease: THE 6th CONFERENCE ON PREVENTION FOR RHEUMATIC FEVER AND RHEUMATIC HEART DISEASE. *Japanese Circulation Journal-english Edition.* 1982;46(11):1239–1242. 10.1253/jcj.46.1239 7131714

[5] Tremoulet AH, Jain S, Jaggi P (2014). Infliximab for intensification of primary therapy for Kawasaki disease: a phase 3 randomised, double-blind, placebo-controlled trial. The Lancet.

[6] Son MB, Gauvreau K, Burns JC (2011). Infliximab for Intravenous Immunoglobulin Resistance in Kawasaki Disease: A Retrospective Study. The Journal of pediatrics.

[7] Sonoda K, Mori M, Hokosaki T, Yokota S (2014). Infliximab Plus Plasma Exchange Rescue Therapy in Kawasaki Disease. The Journal of pediatrics.

[8] Hokosaki T, Mori M, Nishizawa T (2012). Long-term efficacy of plasma exchange treatment for refractory Kawasaki disease. Pediatrics International.

[9] Tremoulet AH, Pancoast P, Franco A (2012). Calcineurin Inhibitor Treatment of Intravenous Immunoglobulin–Resistant Kawasaki Disease. The Journal of pediatrics.

[10] Suzuki H, Terai M, Hamada H (2011). Cyclosporin A Treatment for Kawasaki Disease Refractory to Initial and Additional Intravenous Immunoglobulin. Pediatric Infectious Disease Journal.

[11] Kobayashi T, Inoue Y, Takeuchi K (2006). Prediction of Intravenous Immunoglobulin Unresponsiveness in Patients With Kawasaki Disease. Circulation.

[12] Egami K, Muta H, Ishii M (2006). Prediction of resistance to intravenous immunoglobulin treatment in patients with Kawasaki disease. The Journal of pediatrics.

[13] Sano T, Kurotobi S, Matsuzaki K (2007). Prediction of non-responsiveness to standard high-dose gamma-globulin therapy in patients with acute Kawasaki disease before starting initial treatment. European journal of pediatrics.

[14] Davies S, Sutton N, Blackstock S (2015). Predicting IVIG resistance in UK Kawasaki disease. Archives of disease in childhood.

[15] Rigante D, Andreozzi L, Fastiggi M, Bracci B, Natale MF, Esposito S (2016). Critical Overview of the Risk Scoring Systems to Predict Non-Responsiveness to Intravenous Immunoglobulin in Kawasaki Syndrome. International journal of molecular sciences.

[16] Sleeper LA, Minich LL, McCrindle BM (2011). Evaluation of Kawasaki disease risk-scoring systems for intravenous immunoglobulin resistance. The Journal of pediatrics.

[17] Jakob A, Von Kries R, Horstmann J, et al. Failure to Predict High-Risk Kawasaki Disease Patients in a Population-Based Study Cohort in Germany. Pediatric Infectious Disease Journal. 2018;37(9):850–5. 10.1097/INF.0000000000001923 29406464

[18] Sanchezmanubens J, Anton J, Bou R (2016). Role of the Egami score to predict immunoglobulin resistance in Kawasaki disease among a Western Mediterranean population. Rheumatology international.

[19] Park HM, Lee D, Hyun MC, Lee SB (2013). Predictors of nonresponse to intravenous immunoglobulin therapy in Kawasaki disease. Korean journal of pediatrics.

[20] Fu PP, Du ZD, Pan YS (2013). Novel predictors of intravenous immunoglobulin resistance in Chinese children with Kawasaki disease. Pediatric Infectious Disease Journal.

[21] Sleeper LA, Minich LL, Mccrindle BM (2011). Evaluation of Kawasaki Disease Risk-Scoring Systems for Intravenous Immunoglobulin Resistance. The Journal of pediatrics.

[22] Tremoulet AH, Best BM, Song S (2008). Resistance to Intravenous Immunoglobulin in Children with Kawasaki Disease. The Journal of pediatrics.

[23] Matsubara T, Ichiyama T, Furukawa S (2005). Immunological profile of peripheral blood lymphocytes and monocytes/macrophages in Kawasaki disease. Clinical experimental immunology.

[24] Wang Y, Qian SY, Yuan Y, et al. Do Cytokines Correlate with Refractory Kawasaki Disease in Children. *Clinica Chimica Acta.* 2020. 10.1016/j.cca.2020.03.014 32156603

[25] Mackiewicz A, Speroff T, Ganapathi MK, Kushner I (1991). Effects of cytokine combinations on acute phase protein production in two human hepatoma cell lines. Journal of immunology (Baltimore Md: 1950).

[26] Levitt DG, Levitt M (2016). Human serum albumin homeostasis: a new look at the roles of synthesis, catabolism, renal and gastrointestinal excretion, and the clinical value of serum albumin measurements. International journal of general medicine.

[27] Francharcas G (2001). The meaning of hypoalbuminaemia in clinical practice. Clinical Nutrition.

[28] Yang S, Song R, Zhang J, Li X, Li C (2019). Predictive tool for intravenous immunoglobulin resistance of Kawasaki disease in Beijing. Archives of disease in childhood.

[29] Lin M, Chang C, Sun L (2016). Risk factors and derived formosa score for intravenous immunoglobulin unresponsiveness in Taiwanese children with Kawasaki disease. Journal of The Formosan Medical Association.

[30] Tang Y, Yan W, Sun L (2016). Prediction of intravenous immunoglobulin resistance in Kawasaki disease in an East China population. Clinical rheumatology.

[31] Liu X, Zhou K, Hua Y (2020). Prospective Evaluation of Neutrophil-to-lymphocyte Ratio and Platelet-to-lymphocyte Ratio for Intravenous Immunoglobulin Resistance in a Large Cohort of Kawasaki Disease Patients. The Pediatric infectious disease journal.

[32] Shao S, Luo C, Zhou K (2019). Predictive value of serum procalcitonin for both initial and repeated immunoglobulin resistance in Kawasaki disease: a prospective cohort study. Pediatric rheumatology online journal.

[33] Johnson PJ, Berhane S, Kagebayashi C (2015). Assessment of liver function in patients with hepatocellular carcinoma: a new evidence-based approach -the ALBI grade. J Clin Oncol.

[34] Shimose S, Iwamoto H, Niizeki T, et al. Clinical Significance of Adverse Events for Patients with Unresectable Hepatocellular Carcinoma Treat ed with Lenvatinib: A Multicenter Retrospective Study. Cancers (Basel). 2020;12(7):1867. 10.3390/cancers12071867 PMC7408786 32664489

[35] Miksad R, Cicin I, Chen Y (2019). Outcomes based on Albumin-Bilirubin (ALBI) grade in the phase 3 CELESTIAL trial of cabozantinib versu s placebo in patients with advanced hepatocellular carcinoma (HCC). Annals of oncology: official journal of the European Society for Medical Oncology.

[36] Chan AWH, Zhong J, Berhane S (2018). Development of pre and post-operative models to predict early recurrence of hepatocellular carcinoma after surgical resection. Journal of hepatology.

[37] Wang YY, Zhong JH, Su ZY (2016). Albumin-bilirubin versus Child-Pugh score as a predictor of outcome after liver resection for hepatoc ellular carcinoma. Br J Surg.

[38] Yamamoto K, Honda T, Ito T (2021). The relationship between oral-origin bacteria in the fecal microbiome and albumin-bilirubin grade in patients with hepatitis C. Journal of gastroenterology hepatology.

[39] Shi L, Zhang D, Zhang J (2020). Albumin-bilirubin score is associated with in-hospital mortality in critically ill patients with acute pancreatitis. Eur J Gastroenterol Hepatol.

[40] Nakajima T, Karino Y, Hige S (2021). Factors affecting the recovery of hepatic reserve after sustained virologic response by direct-acting antiviral agents in chronic hepatitis C virus-infected patients. Journal of gastroenterology hepatology.

[41] Bayers S, Shulman ST, Paller AS. Kawasaki disease: Part II. Complications and treatment. J Am Acad Dermatol*.* 2013;69(4):513.e511-513.e518. 10.1016/j.jaad.2013.06.040 24034380

[42] Bayers S, Shulman ST, Paller AS (2013). Kawasaki disease: part I. Diagnosis, clinical features, and pathogenesis. J Am Acad Dermatol.

[43] Jenson HB, Behrman RE, Kliegman R. Nelson Textbook of Pediatrics e-dition 18ED. *Saunders.* 2007.

[44] McCrindle BW, Rowley AH, Newburger JW, et al. Diagnosis, Treatment, and Long-Term Management of Kawasaki Disease: A Scientific Statement for Health Professionals From the American Heart Association. Circulation. 2017;135(17):e927–99. 10.1161/CIR.0000000000000484 28356445

[45] Haycock GB, Schwartz GJ, Wisotsky DH. Geometric method for measuring body surface area: A height-weight formula validated in infants, children, and adults†. The Journal of pediatrics. 1978;93(1):62–6. 10.1016/s0022-3476(78)80601-5 650346

[46] Saji T, Arakaki Y, Fuse S (2016). A New Z -Score Curve of the Coronary Arterial Internal Diameter Using the Lambda-Mu-Sigma Method inaPediatric Population. Journal of the American Society of Echocardiography.

[47] Rimini M, Rovesti G, Casadei-Gardini A (2020). Child Pugh and ALBI grade: past, present or future?. Ann Transl Med.

[48] Feng D, Wang M, Hu J (2020). Prognostic value of the albumin-bilirubin grade in patients with hepatocellular carcinoma and other liver diseases. Ann Transl Med.

[49] Deng M, Ng SWY, Cheung ST, Chong CCN (2020). Clinical application of Albumin-Bilirubin (ALBI) score: The current status. Surgeon.

[50] Huo TI (2019). ALBI grade as a new player in hepatocellular carcinoma. Journal of the Chinese Medical Association: JCMA.

[51] Hua W, Sun Y, Wang Y (2017). A new model to predict intravenous immunoglobin-resistant Kawasaki disease. Oncotarget.

[52] Moon KP, Kim BJ, Lee KJ (2016). Prediction of nonresponsiveness to medium-dose intravenous immunoglobulin (1 g/kg) treatment: an effe ctive and safe schedule of acute treatment for Kawasaki disease. Korean journal of pediatrics.

[53] Tan X, Zhang X, Wang X (2019). A new model for predicting intravenous immunoglobin-resistant Kawasaki disease in Chongqing: a retrospective study on 5277 patients. Scientific reports.

[54] Qian W, Tang Y, Yan W, Sun L, Lv H (2018). A comparison of efficacy of six prediction models for intravenous immunoglobulin resistance in Kawasaki disease. Italian journal of pediatrics.

[55] Fukazawa R, Kobayashi J, Ayusawa M, Hamada H, Kimura T. JCS/JSCS 2020 Guideline on Diagnosis and Management of Cardiovascular Sequelae in Kawasaki Disease. Circulation Journal. 2020. 10.1253/circj.CJ-19-1094 32641591

[56] Chantasiriwan N, Silvilairat S, Makonkawkeyoon K, Pongprot Y, Sittiwangkul R (2018). Predictors of intravenous immunoglobulin resistance and coronary artery aneurysm in patients with Kawasaki disease. Paediatrics international child health.

[57] Kim T, Choi W, Woo CW (2007). Predictive risk factors for coronary artery abnormalities in Kawasaki disease. European journal of pediatrics.

