# Supplementary material for: Predictive value of Albumin-Bilirubin grade for intravenous immunoglobulin resistance in a large cohort of patients with Kawasaki disease: a prospective study
Source: Pediatr Rheumatol Online J. 2021 Sep 25;19:147. doi: 10.1186/s12969-021-00638-7 (PMC8467146; doi:10.1186/s12969-021-00638-7)
Supplement: Supplementary file 1 — Additional file 1: Supplementary material 1. [file 12969_2021_638_MOESM1_ESM.docx]

**Supplemental material 1.** Comparison of clinical data between the groups of repeated IVIG resistance and responsive in KD

|  | **IVIG-responsive**  **(n=70)** | **IVIG-resistance**  **(n=45)** | ***p*-value** |
| --- | --- | --- | --- |
| Male | 35(50.0) | 23(51.1) | 0.560 |
| Age, years | 2.8(1.3-4.7) | 1.2(1.8-4.0) | 0.270 |
| **Clinical manifestations** |  |  |  |
| Rash | 56(80.0) | 40(88.9) | 0.304 |
| Extremity changes | 41(58.6) | 24(53.3) | 0.565 |
| Conjunctivitis | 61(87.1) | 41(91.1) | 0.761 |
| Oral changes | 63(90.0) | 44(97.8) | 0.242 |
| Cervical lymphadenopathy | 39(55.7) | 23(51.1) | 0.701 |
| Fever duration before initial IVIG, days | 5.0(5.0-6.0) | 5.0(4.0-6.0) | 0.322 |
| Incomplete KD | 22(31.4) | 14(31.1) | 1.000 |
| CALs | 10(14.3) | 5(11.1) | 0.779 |
| **Before initial IVIG** |  |  |  |
| WBC, ×10^9^/L | 14.0(10.8-16.4) | 8.5(12.9-18.3) | 0.946 |
| Neutrophil, % | 77.3(67.6-83.9) | 77.7(60.3-85.9) | 0.426 |
| Lymphocyte, % | 17.0(10.4-23.0) | 12.8(7.9-26.4) | 0.735 |
| Hemoglobin, g/L | 109.0(102.0-115.0) | 103.0(96.5-111.5) | 0.013 |
| PLT, ×10^9^/L | 304(228-363) | 280(196-333) | 0.231 |
| CRP, mg/L | 83.0(60.5-118.5) | 118.0(69.0-160.0) | 0.019 |
| ESR, mm/h | 67.0(48.0-88.0) | 67.0(46.0-82.0) | 0.955 |
| AST, U/L | 38.0(24.5-89.5) | 34.0(25.0-58.0) | 0.241 |
| ALT, U/L | 79.0(26.0-146.5) | 46.0(26.5-95.0) | 0.143 |
| ALB, g/L | 37.0(32.0-39.0) | 34.0(29.0-38.2) | 0.038 |
| TBil, μmol/L | 7.7(5.6-17.3) | 7.0(4.0-31.2) | 0.236 |
| Na^+^, mmol/L | 135.4(133.4-137.0) | 134.0(131.3-136.0) | 0.033 |
| ALBI | –2.47[–2.8-(–2.02)] | –2.27[–2.64-(–1.9)] | 0.081 |

Abbreviations: ALB, Albumin; AST, aspartate aminotransferase; ALT, alanine aminotransferase; BMI, body mass index; CRP, C-reactive protein; CAR, C-reactive protein-to-albumin ratio; CALs, coronary artery lesions; ESR, erythrocyte sedimentation rate; IVIG, intravenous immunoglobulin; KD, Kawasaki disease; NLR, neutrophil-lymphocyte ratio; PLR, platelet-lymphocyte ratio; TBil, total bilirubin; Na^+^, sodium; WBC, white blood cell;

The data are presented as the median with the 25th and 75th percentiles in square brackets for continuous variables and as the percentage for the categorical variables
