# Supplementary material for: Predictive value of Albumin-Bilirubin grade for intravenous immunoglobulin resistance in a large cohort of patients with Kawasaki disease: a prospective study
Source: Pediatr Rheumatol Online J. 2021 Sep 25;19:147. doi: 10.1186/s12969-021-00638-7 (PMC8467146; doi:10.1186/s12969-021-00638-7)
Supplement: Supplementary file 2 — Additional file 2: Supplementary material 2. [file 12969_2021_638_MOESM2_ESM.docx]

**Supplementary material 2.** Comparison of clinical data between the groups of Cals and non-CALs in patients with KD

|  | **Patients with CALs**  **(n=86)** | **Patients with**  **non-CALs**  **(n=737)** | ***p* value** |
| --- | --- | --- | --- |
| Male | 61(70.9) | 401(54.4) | 0.004 |
| Age, years | 2.5(1.1-4.4) | 2.2(1.2-3.6) | 0.101 |
| **Clinical manifestations** |  |  |  |
| Rash | 65(78.6) | 582(79.0) | 0.487 |
| Extremity changes | 44(51.2) | 415(56.3) | 0.422 |
| Conjunctivitis | 77(89.5) | 103(89.6) | 0.368 |
| Oral changes | 70(81.4) | 677(91.9) | 0.005 |
| Cervical lymphadenopathy | 38(44.2) | 310(42.1) | 0.730 |
| Fever duration before initial IVIG, days | 5.0(4.8-7.0) | 5.0(5.0-6.0) | 0.485 |
| Incomplete KD | 42(48.8) | 269(36.5) | 0.034 |
| IVIG resistance | 15(17.4) | 100(13.6) | 0.325 |
| **Before initial IVIG** |  |  |  |
| WBC, ×10^9^/L | 13.4(10.5-17.5) | 13.3(10.7-16.6) | 0.502 |
| Neutrophil, % | 69.6(59.5-79.7) | 67.7(56.9-77.8) | 0.334 |
| Lymphocyte, % | 21.1(13.4-30.2) | 23.1(15.0-32.2) | 0.247 |
| Hemoglobin, g/L | 107.5(101.0-114.3) | 109.0(102.0-117.0) | 0.400 |
| PLT, ×10^9^/L | 311.0(209.8-409.5) | 313.0(255.0-377.0) | 0.985 |
| CRP, mg/L | 79.3(48.8-124.5) | 73.0(43.1-111.0) | 0.223 |
| ESR, mm/h | 59.5(44.0-85.5) | 65.0(46.0-81.0) | 0.804 |
| AST, U/L | 31.0(24.0-46.7) | 33.0(25.0-50.5) | 0.361 |
| ALT, U/L | 34.5(18.5-78.0) | 38.0(21.0-88.0) | 0.457 |
| ALB, g/L | 37.0(33.0-40.2) | 38.0(41.0-41.0) | 0.034 |
| TBil, μmol/L | 5.8(4.0-8.8) | 6.0(4.0-9.0) | 0.981 |
| Na^+^, mmol/L | 137.0(134.0-140.0) | 137.0(134.0-139.0) | 0.334 |
| ALBI | –2.63(–2.92- (–2.19)) | –2.70(–3.00- (–2.42)) | 0.065 |

Abbreviations: ALB, Albumin; AST, aspartate aminotransferase; ALT, alanine aminotransferase; CRP, C-reactive protein; CALs, coronary artery lesions; ESR, erythrocyte sedimentation rate; IVIG, intravenous immunoglobulin; KD, Kawasaki disease; TBil, total bilirubin; Na^+^, sodium; WBC, white blood cell; ALBI, albumin-bilirubin index;

The data are presented as the median with the 25th and 75th percentiles in square brackets for continuous variables and as the percentage for the categorical variables.
