# Supplementary material for: Predictive value of Albumin-Bilirubin grade for intravenous immunoglobulin resistance in a large cohort of patients with Kawasaki disease: a prospective study
Source: Pediatr Rheumatol Online J. 2021 Sep 25;19:147. doi: 10.1186/s12969-021-00638-7 (PMC8467146; doi:10.1186/s12969-021-00638-7)
Supplement: Supplementary file 3 — Additional file 3: Supplementary material 3. [file 12969_2021_638_MOESM3_ESM.docx]

**Supplementary material 3** Sensitivity, specificity, PPV, NPV, diagnostic accuracy of conventional parameters in initial IVIG resistance prediction

|  | AUC | SE | 95%CI | Sensitivity | Specificity | PPV | NPV | Diagnostic accuracy | *p* value |
| --- | --- | --- | --- | --- | --- | --- | --- | --- | --- |
| PLT≤312×10^9^/L | 0.604 | 0.0279 | 0.549-0.659 | 0.635 | 0.521 | 0.177 | 0.898 | 0.537 | <0.001 |
| CRP≥ 57.4 mg/L | 0.617 | 0.0284 | 0.562-0.673 | 0.809 | 0.398 | 0.179 | 0.928 | 0..456 | <0.001 |
| N%≥ 76.2 % | 0.677 | 0.0278 | 0.623-0.731 | 0.530 | 0.733 | 0.244 | 0.906 | 0.705 | <0.001 |
| ALT≥41U/L | 0.606 | 0.0289 | 0.549-0.662 | 0.617 | 0.558 | 0.185 | 0.900 | 0.566 | <0.001 |
| AST≥56 U/L | 0.541 | 0.0319 | 0.478 - 0.603 | 0.330 | 0.802 | 0.213 | 0.881 | 0.736 | <0.001 |
| Na^+^≤135.4mmol/L | 0.687 | 0.0319 | 0.637-0.738 | 0.591 | 0.708 | 0.247 | 0.914 | 0.691 | <0.001 |
| Age≥4.41 years | 0.554 | 0.0307 | 0.494-0.614 | 0.252 | 0.856 | 0.221 | 0.876 | 0.722 | <0.001 |

Abbreviations: ALT, alanine aminotransferase; AST, aspartate aminotransferase; N%, neutrophil percentage; CRP, C-reactive protein; PLT, platelet; Na^+^, sodium; IVIG, intravenous immunoglobulin; NPV, negative predictive value; PPV, positive predictive value.
