# Supplementary material for: Predictive value of Albumin-Bilirubin grade for intravenous immunoglobulin resistance in a large cohort of patients with Kawasaki disease: a prospective study
Source: Pediatr Rheumatol Online J. 2021 Sep 25;19:147. doi: 10.1186/s12969-021-00638-7 (PMC8467146; doi:10.1186/s12969-021-00638-7)
Supplement: Supplementary file 4 — Additional file 4: Supplementary material 4. [file 12969_2021_638_MOESM4_ESM.docx]

**Supplementary material 4.** The sensitivity, specificity, PPV and NPV of all available risk-scoring systems for IVIG resistance prediction in our population

| Risk scoring-systems | Sensitivity | Specificity | PPV | NPV |
| --- | --- | --- | --- | --- |
| Kobayashi | 0.400 | 0.860 | 0.317 | 0.898 |
| Egami | 0.435 | 0.727 | 0.206 | 0.888 |
| Sano | 0.365 | 0.845 | 0.276 | 0.891 |
| Formosa | 0.661 | 0.575 | 0.202 | 0.913 |
| Moon’s | 0.670 | 0.621 | 0.223 | 0.921 |
| Fu’s | 0.617 | 0.636 | 0.216 | 0.911 |
| Tang’s | 0.348 | 0.838 | 0.258 | 0.888 |
| Yang’s | 0.513 | 0.756 | 0.254 | 0.905 |
| Hua’s | 0.170 | 0.950 | 0.41 | 0.880 |
